# Another Case of Bell's Palsy Recurrence After Pfizer-BioNTech COVID-19 Vaccination

**DOI:** 10.7759/cureus.27422

**Published:** 2022-07-28

**Authors:** Huailin Zhang, Diana Sanchez Gomez, Michael Repajic, Antonio K Liu

**Affiliations:** 1 Internal Medicine, Adventist Health White Memorial, Los Angeles, USA; 2 Family Medicine, Adventist Health White Memorial, Los Angeles, USA; 3 Neurology, University of Southern California Keck School of Medicine, Los Angeles, USA; 4 Neurology, Adventist Health White Memorial, Los Angeles, USA; 5 Neurology, Loma Linda University School of Medicine, Loma Linda, USA

**Keywords:** safety, recurrence, bell's palsy, covid vaccination, pandemic

## Abstract

Twenty-seven months into the current pandemic and 18 months after vaccinations were made available, there are still relatively limited data on the incidence of recurrent Bell's Palsy after the administration of mRNA-based vaccines. The authors continue to believe that it is through rigorous reporting that the true incidence can be tabulated eventually.

## Introduction

In both the Moderna and Pfizer-BioNTech coronavirus disease 2019 (COVID-19) vaccine clinical trials conducted at 99 centers across the United States, Bell’s Palsy was listed as a possible side effect [1]. After reporting a case of Bell’s Palsy recurrence in a patient who received the Pfizer-BioNTech COVID-19 vaccine [2], we encountered another patient with recurrence of Bell's Palsy six days after the administration of the first dose of the Pfizer-BioNTech COVID-19 vaccine. This report intends to add to the growing literature regarding the safety profile of this new vaccine.

## Case presentation

**Figure 1:**
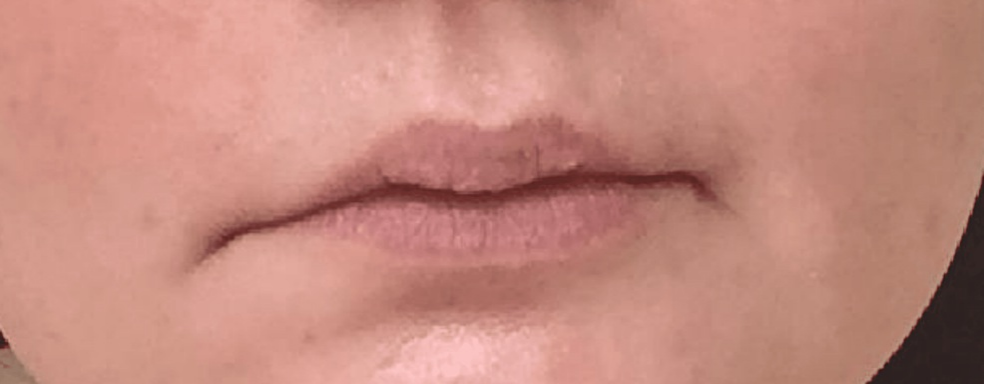
Patient with right facial droop.

**Figure 2:**
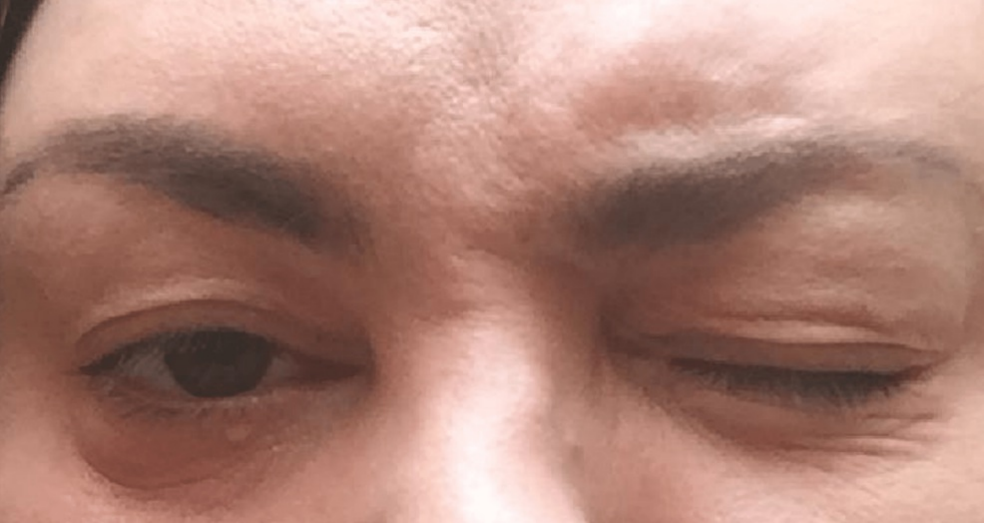
Patient unable to close her right eye.

The patient is a 38-year-old female who connected with us upon reading our previous report. She had been previously healthy except for a prior episode of left-sided Bell’s Palsy around 10 years earlier. The symptoms from her first episode were described as severe with eventual full recovery after receiving a combination of a brief course of prednisone, antiviral medication, and acupuncture. The patient received her first dose of the Pfizer-BioNTech COVID-19 vaccine on April 29th of 2021 and began to experience tongue numbness six days later. The following day, she developed a right facial droop (Figures 1-2). The rest of her neurological examination was negative and her workup was unremarkable. Her symptoms progressed over the next few days, which prompted treatment with a week-long course of prednisone. Serial follow-up exams revealed a slower improvement in her symptomatology in comparison to her episode 10 years ago.

## Discussion

This report marks another episode of Bell’s Palsy recurrence following administration of the mRNA COVID-19 vaccination that the same practice has encountered this year, suggesting a potential association between the mRNA vaccine and these recurrences. COVID infection as well as mRNA-based vaccines have been associated with many neurological conditions, including Bell’s Palsy [3]. Data on recurrent Bell's Palsy are lacking; our previously reported Bell's Palsy recurrence after mRNA-based vaccine remains the only peer-reviewed case report in the literature [2]. Some have suggested that type I interferons, which are strongly elicited by mRNA vaccinations, may play a contributory role. Several case reports have hypothesized that a similar phenomenon may be contributing to the development of Bell’s Palsy after hepatitis C virus treatment as well as administration of another mRNA vaccine [4-6]. Additionally, at the molecular level, activation of the p53 upregulated modulator apoptosis (PUMA) and innate immunity signaling module (SARM1) can lead to axonal degeneration [7]. Another study suggested that upregulation of the aquaporin 1 water channel protein can lead to infratemporal facial nerve edema and eventual impingement in the temporal bone canal [8].

## Conclusions

With the wide roll-out of mRNA COVID-19 vaccines, population-level surveillance will be required to establish any concrete association between recurrent Bell’s palsy and the vaccines. Possible adverse reactions such as the one described here should be diligently reported to help establish a more reliable vaccine safety profile. With this documentation, we hope to help reassure individuals with doubts about vaccination that such occurrences are still very low.
